# University of Maryland Dental Department

**Published:** 1885-10

**Authors:** 


### Editorial, Etc.

University of Maryland, Dental Department.—The fourth Annual session of this institution opened with a much larger number of matriculates than ever before in its history, and the number is so rapidly increasing that the present class of seniors and juniors bids fair to be larger than any preceding one.

The reputation of this school has never been sullied by the graduation of students for fees irrespective of professional ability, and the consequence of such a course as has been steadily pursued since its organization, has been to give a professional standing to its diploma which that of no other dental school excels. The present class consists of representatives from all parts of this country, and also Germany, France, South America, Canada, and even Turkey. Many states of this country are largely represented, such as New York, Georgia, Virginia, Pennsylvania, South Carolina and Maryland especially, and also the New England states, while nearly every other state is represented.

Students who have passed a session at other dental schools have entered on a second session at the University of Maryland, Dental Department, to complete their course of study and receive its diploma, and not one of the hundreds of students who have attended a course in this institution, has ever gone elsewhere to graduate. In matriculating the present class, the resolutions adopted by the National Board of State Dental Examiners have been strictly adhered to, and many applicants of this country and Europe have been refused admission who desired to make their attendance obligatory on graduation after ONE session's attendance.

The Infirmary and Labratory practice is not exceled in size if equalled by that of any other dental school, and the records will show hundreds of gold fillings credited to the individual practice of students for both the regular winter and summer sessions. No other school can offer greater facilities for practical instruction, nor present more complete equipments as to building and appliances than this Dental Department. Dental practitioners are cordially invited to visit the University and inspect the specimen work of its graduating classes deposited in the museum. Large and valuable contributions from all parts of this country and also from Europe are almost daily being received for the Museum, which will compare favorably with that of any other dental school for valuable pathological specimens, which are also utilized for illustrating the lectures of each course.

